# Clasp and Bar Retention

**Published:** 1913-09-15

**Authors:** S. F. Gilmore


### CLASP AND BAR RETENTION.

Dr. G. V. Black demonstrated years ago, by systematic experiment, that a vigorous use of the teeth in masticating, together with well-conducted prophylactic methods, had a tendency to bring an otherwise lame tooth, barely able to withstand a bite pressure of forty or fifty pounds, to a condition of efficiency that enabled its owner to exert a force of 170 pounds, illustrating beyond cavil that enforced pressure on the supporting fibers of a tooth cannot but have a salutary effect, provided, of course, that the force comes from an angle contemplated by nature.

I am not presuming that the foregoing remarks present anything with which you are not all familiar. I simply offer them as preliminary to an explanation and illustrations of a system of anchorage and retention for dentures that, under favorable conditions, may be listed as a practical mechanical method of replacing organs that have been lost through the ravages of caries, trauma, and adverse pathological conditions in general. In view of the fact that the alveolar ridge is called upon to bear a part of the burden of supporting the artificial substitute, the method may be called a transition from bridge to plate prosthesis. There is nothing new or novel connected with employing a natural tooth or root as an anchor for artificial teeth, and at the same time afford the wearer an opportunity to remove and replace the denture. In fact, within the past decade a number of such methods have been exploited, all of them possessing merit, but limited in a measure to conditions; and while the systems provide for employment of the ridge as a support, the element of crown leverage over the root is not sufficiently eliminated. Then, too, if the patient is so unfortunate as to have nothing left in the shape of a natural tooth, but one or two roots, it was impossible to use them without resorting to an artificial crown, with its inevitable lever action, over the root, while the method under consideration enables the operator to avail himself (to the intense comfort and gratification of his patient) of even one root to act as an anchor no matter what its location may be, in either the maxilla or mandible. A pleasing esthetic effect accompanies the employment of a root or roots, in this, that the retaining mechanism is entirely concealed by the denture, and not even accessible to the tongue or sense of touch by the patient. When the angle bar is attached to a shell crown or porcelain crown with metallic base, the question has arisen as to the probable effect the lever action of the bar might have on the tooth.

Replying to this, I may say that I have no data on the subject covering a period longer than eight years. However, conditions in these cases were so satisfactory that I am led to believe we are justified in adopting the method in all cases where its application is indicated, because I am convinced by clinical experience that the question of injurious results is a negligible one, so far as the lever action of the angle bar is concerned. It must be conceded that in a distal extension, involving the second bicuspid and first and second molars, if shrinkage of the ridge takes place, pressure will accumulate on the bar, with resultant movement of the anchor tooth to accommodate itself to changed conditions, and from a mechanical point of reasoning, should throw the tooth out of its normal perpendicular. To eliminate guesswork and arrive at satisfactory conclusions along this line, the late Dr. John Q. Byram found, by laboratory experiment, that if an extension, such as the one above mentioned, settled one-eighth of an inch at its distal extremity, the axial line of the anchor tooth would only be compelled to yield one-sixty-fourth of an inch from normal. Now, if the orthodontist is to be congratulated on performing extensive tooth movements, the general practitioner should, as Dr. C. E. Mills says, "be immune to criticism for causing this limited change of position, unaccompanied by any injurious results."

The lateral stress against the bar is minimized by the adaptation of the denture to the remaining teeth and uneven surfaces of the ridge, so that, taking it all in all, I feel safe in saying that, from my own experience and that of others, we may, with propriety, offer the adjustable attachment method to our patients.

It goes without saying that the practicability of the application and the indication for the employment of the method, can only be determined through a careful consideration of the conditions presented in each case.

The dental profession is in the lime light at this time, in the matter of operative procedures. Certain members of the medical profession have epitomized our extensive fixed replacements as unsanitary, alleging that we have created inaccessible pockets and hiding places that defy the most strenuous efforts on the part of the patient to comply with the rules of oral hygiene. We are loath to admit the truth of the allegation, and still conscious of the fact that in many cases we have slim grounds for defense.—S. F. Gilmore, in *Journal of Allied Societies.*

